# Intra-arterial transplantation of stem cells in large animals as a minimally-invasive strategy for the treatment of disseminated neurodegeneration

**DOI:** 10.1038/s41598-021-85820-3

**Published:** 2021-03-22

**Authors:** Izabela Malysz-Cymborska, Dominika Golubczyk, Lukasz Kalkowski, Joanna Kwiatkowska, Michal Zawadzki, Joanna Głodek, Piotr Holak, Joanna Sanford, Kamila Milewska, Zbigniew Adamiak, Piotr Walczak, Miroslaw Janowski

**Affiliations:** 1 Department of Neurosurgery, School of Medicine, Collegium Medicum, University of Warmia and Mazury, Olsztyn, Poland; 2 Central Clinical Hospital of Ministry of the Interior and Administration in Warsaw, Warsaw, Poland; 3 Department of Surgery and Radiology, Faculty of Veterinary Medicine, University of Warmia and Mazury, Olsztyn, Poland; 4 Sanford Biotech, Warsaw, Poland; 5 Center for Advanced Imaging Research and Department of Diagnostic Radiology and Nuclear Medicine, University of Maryland School of Medicine, Baltimore, MD USA

**Keywords:** Neuroscience, Diseases of the nervous system, Regeneration and repair in the nervous system, Stem cells in the nervous system

## Abstract

Stem cell transplantation proved promising in animal models of neurological diseases; however, in conditions with disseminated pathology such as ALS, delivery of cells and their broad distribution is challenging. To address this problem, we explored intra-arterial (IA) delivery route, of stem cells. The goal of this study was to investigate the feasibility and safety of MRI-guided transplantation of glial restricted precursors (GRPs) and mesenchymal stem cells (MSCs) in dogs suffering from ALS-like disease, degenerative myelopathy (DM). Canine GRP transplantation in dogs resulted in rather poor retention in the brain, so MSCs were used in subsequent experiments. To evaluate the safety of MSC intraarterial transplantation, naïve pigs (n = 3) were used as a pre-treatment control before transplantation in dogs. Cells were labeled with iron oxide nanoparticles. For IA transplantation a 1.2-French microcatheter was advanced into the middle cerebral artery under roadmap guidance. Then, the cells were transplanted under real-time MRI with the acquisition of dynamic T2*-weighted images. The procedure in pigs has proven to be safe and histopathology has demonstrated the successful and predictable placement of transplanted porcine MSCs. Transplantation of canine MSCs in DM dogs resulted in their accumulation in the brain. Interventional and follow-up MRI proved the procedure was feasible and safe. Analysis of gene expression after transplantation revealed a reduction of inflammatory factors, which may indicate a promising therapeutic strategy in the treatment of neurodegenerative diseases.

## Introduction

The central nervous system (CNS) is by far the most challenging therapeutic target, and its endogenous regenerative capacity is minimal. Usually the prognoses of patients suffering from neurological disorders are dire, elevating pressure to develop new therapeutic strategies. One of the most controversial approaches of the last several decades has been the use of stem cells^1,2^. The potential of stem cells has enthralled the world, but more than 30 years after the first clinical trials, outcomes are rather disappointing. This is despite impressive progress with the identification of compelling therapeutic targets such as replacement of glia^3^ or isolation of highly potent stem cell sources such as primary neural stem cells^4^. There is a growing consensus that the reason behind the suboptimal therapeutic effect of otherwise highly potent stem cells is their inadequate delivery. There are several gateways to the brain, but the most common route of stem cell administration is intravenous (IV) infusion^5,6^. While easy to perform, non-invasive and safe, the IV route has proven to result in very low efficacy of cell accumulation in the brain^7^. To address these shortcomings, the intra-arterial (IA) route has been utilized as much more effective, taking advantage of the first pass trapping effect resulting in excellent cell accumulation in the brain^8^. Over the last decade significant advances have been made using targeted IA delivery, including robust technology for image-guidance to improve reproducibility, precision and safety of targeted IA administration^9,10^. Real-time image-guided MRI targeting of therapeutic agents enables delivering therapy to a selected region of the brain with high accuracy and reproducibility. Unique to IA administration, therapeutics can be distributed at high concentrations, broadly and uniformly throughout the targeted brain territory, and with minimal systemic exposure^11^. Image-guided targeting of cells globally to the entire central nervous system creates the opportunity to make significant advancements in developing a breakthrough treatment for diseases with disseminated pathology such as amyotrophic lateral sclerosis (ALS). The most common approach to pre-clinical modeling of ALS are genetic rodent models^12,13^. While they represent a valuable and cost-effective strategy for the initial screening of therapeutic compounds, a direct translation of outcomes from small animals to humans led to many failed clinical studies. Large animals are important in modeling neurological diseases because of their high clinical relevance (e.g. gyrencephalic, large brain with a high proportion of white-to-gray matter ratio), another advantage is the suitability of clinical imaging modalities for monitoring disease course and therapies^14^. Moreover, thanks to a larger size blood vessels, endovascular procedures can be easily applied. With a larger brain size and closer to the human immune system, the assessment of stem cell transplantation is also more reliable^15,16^. Therefore, we decided to use a unique human ALS model—dogs with naturally occurring degenerative myelopathy (DM)^3,4^. The size of the brain, the environment shared with humans, and the natural occurrence of the disease are more representative of human conditions and, as such, provide an excellent model that bridges rodent and clinical studies.

Moreover, the size and the cerebral vasculature of the dog brain enables navigation of IA catheters towards distal cerebral vessels and targeted delivery of stem cells under the guidance of clinical imaging equipment, further improving clinical relevance. Considering the advantages mentioned above of the image-guided IA route, we utilized this strategy to deliver stem cells directly to the brain in dogs suffering from DM. The primary goal of this study was to show the feasibility and safety of image-guided IA delivery route to the brain. As therapeutic agents, we used two major classes of stem cells: glial restricted precursors (GRPs), and mesenchymal stem cells (MSCs). During the development of the central nervous system, GRPs give rise to oligodendrocytes and astrocytes^17^. Therefore, they are considered as good cell candidates for the treatment of neurodegenerative diseases. GRPs implanted into the adult spinal cord exhibit robust survival, proliferation and migration as well as differentiation into mature glial phenotypes. GRPs have been shown to produce myelin, help reduce glial scar formation, promote axonal growth and regeneration, and support synaptic transmission^18–20^. In rodent models of ALS, GRPs have revealed strong therapeutic effect prolonging lifespan of animals and improving motor functions^13,21^. As multipotent stem cells, MSCs, can differentiate into various types of cells, e.g. chondrocytes, myocytes, adipocytes etc. Therefore, for many years they have been broadly used to develop the treatment of neurological disorders^22,23^. Notably, when co-transplanted with GRPs, MSCs improved survival of GRPs, through secreting anti-inflammatory and trophic factors in mice^24^. Given the above and the etiology of DM, we believe that the evaluation of the therapeutic potential of both GRPs and MSCs after their targeted intra-arterial administration to the brain is warranted.

## Results

### Intra-arterial MRI-guided transplantation of canine GRPs (cGRPs) in DM dogs

**Figure 1:**
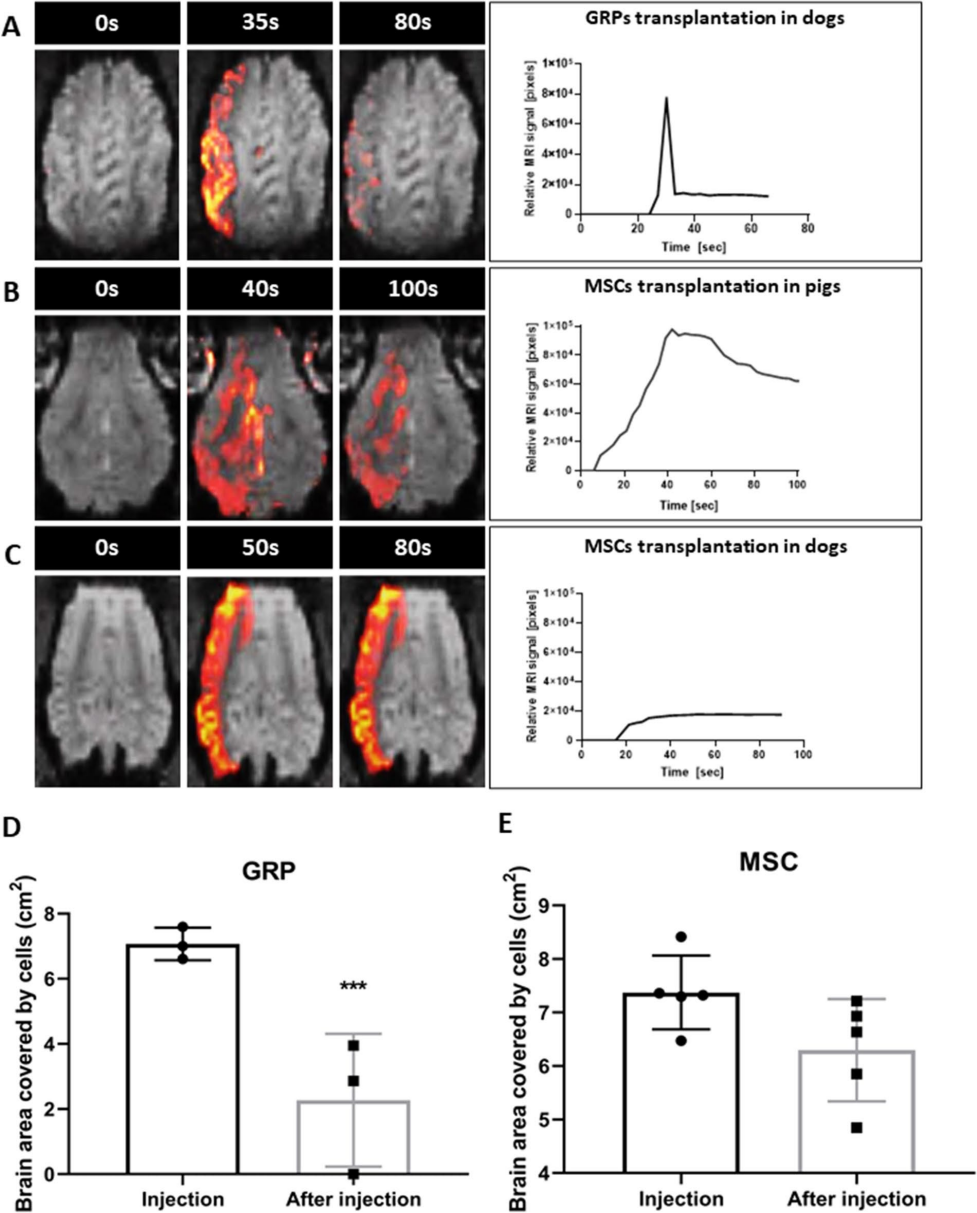
MRI-guided transplantation of stem cells. (**A**) MRI-guided intraarterial transplantation of SPION labeled canine GRPs in DM dogs. (**B**) MRI-guided intraarterial transplantation of SPION labeled porcine MSCs in pig. (**C**) MRI-guided intraarterial transplantation of SPION canine MSCs in DM dogs. (**D**) Brain area covered by transplanted GRP cells during and post injection in DM dogs. (**E**) Brain area covered by transplanted MSC cells during and post injection in DM dogs. Data are expressed as mean with standard deviation (SD) and differences were considered as statistically significant at the 95% confidence level (***P < 0.001).

Dogs with their internal carotid artery cannulated under X-ray angiography were placed in 3 T MRI (Magnetom-Trio, Siemens) for MRI-guided injection. Prior to cell transplantation, trans-catheter perfusion territory was visualized using the IA injection of Feraheme contrast (AMAG pharmaceuticals, Waltham, USA) and the infusion rate was adjusted to achieve adequate coverage throughout the ipsilateral hemisphere. cGRPs were injected IA while acquiring dynamic GE-EPI scans using the infusion speed that was predetermined by contrast agent pre-injection. During cGRP infusion, signal intensity change was observed in the ipsilateral cortex, indicating labeled cells entered cortical circulation (Fig. 1A). Quantitative analysis of the dynamic scans revealed that the maximum signal was found at the end of infusion (35 s). Immediately upon infusion completion, it rapidly cleared, suggesting that cells were not captured by cerebral endothelium and perfused out (Fig. 1A,D). Brain territory with a hypointense signal indicating localization of cGRP cells during vs. after transplantation was 7.02 ± 0.39 cm^2^ and 2.27 ± 2.04 cm^2^, respectively, which confirms that the brain area covered with cells had been reduced by 74.11% (Fig. 1A; *P* < 0.001).

### Intra-arterial MRI-guided transplantation of porcine MSCs (pMSCs) in pigs

**Figure 2:**
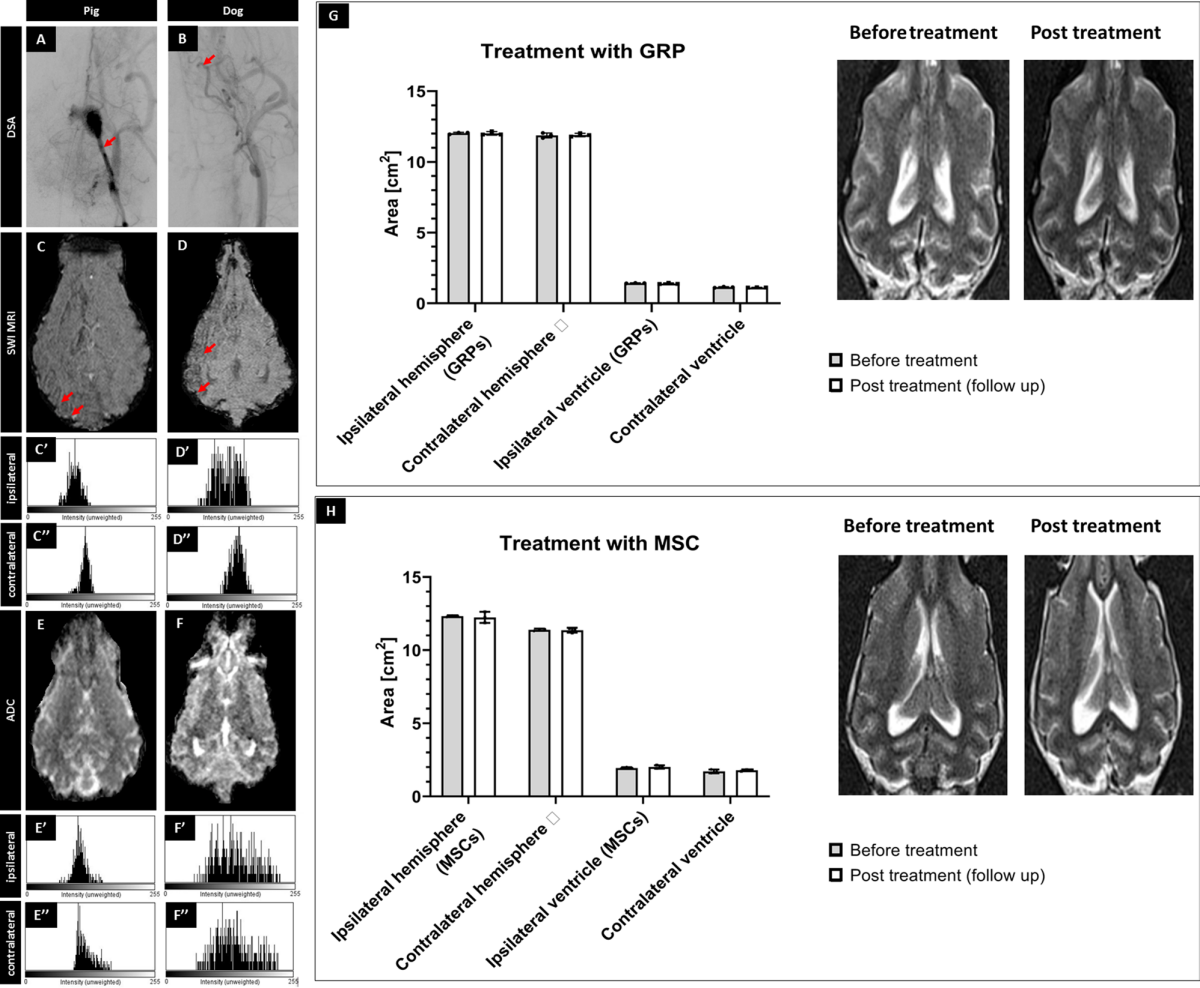
Safety of the procedures. X-ray evaluation of catheter placement (**A**,**B**; arrows indicate placement of the tip of microcatheter). SWI (**C**,**D**) with histograms from ipsilateral (**C′**,**D′**) and contralateral (**C″**,**D″**) hemisphere showing the accumulation of hypointense pixels in the ipsilateral hemisphere. ADC scans (**E**,**F**) after pig's and canine's MSC transplantation with histograms from ipsilateral (**E′**,**F′**) and contralateral (**E″**,**F″**) hemisphere showing any changes in diffusion after cell transplantation. Evaluation of size of hemispheres and ventricles visible on the MRI before versus post-transplantation of GRPs (**G**) and MSCs (**H**) showing no differences in size before and after transplantation.

IA transplantation of small cGRPs in dogs showed weak cell homing in the brain; thus, we decided to use MSCs, which are more significant and known for better homing. MSCs have been associated with microembolic complications; hence before their use in companion DM dogs, the safety of the selected dose was tested in pigs. IA catheter was placed under X-ray angiography in the pharyngeal ascendant artery just proximal to rete (Fig. 2A). Dynamic MRI during cell infusion revealed extensive signal intensity change throughout the ipsilateral hemisphere, delineating flow and distribution of SPION-labeled MSCs (Fig. 1B). The hypointense signal of pMSCs at the end of the injection, expressed in a relative number of pixels, decreased only by 31.25 ± 2.10% compared to the initial value (Fig. 1B). This indicates better endothelial capture and settlement of MSCs in the brain following IA pMSC transplantation.

### Real-time MRI monitored cMSCs intra-arterial transplantation in DM dogs

Based on data obtained during MSC transplantation in pigs, we proceeded to transplant canine MSCs in DM dogs. The cMSCs labeled with SPION were injected with the same rate as in pigs, when the dog was inside the MRI scanner with dynamic imaging showing cogent accumulation of cells throughout the ipsilateral hemisphere (Figs. 1C and 2B). There was a gradual accumulation of the hypointense pixels with the maximum signal detected towards the end of cMSCs’ infusion. Cells covered 7.37 ± 0.31 cm^2^ of hemisphere during injection and 6.30 ± 0.427 cm^2^ at the end of the infusion, which means that clearance of cells after completed injection was only 15% (Fig. 1C,E). This indicates that cMSCs were captured very well by cerebral endothelium.

### Safety of intra-arterial cell transplantation

It has been shown that the IA injection of MSCs is associated with the risk of microembolism^25^ when an excessive number of cells are infused. To examine the safety of transplantation, we performed diffusion scans, particularly focusing on regions with high cell uptake and did not observe any focal or diffuse abnormalities on SWI and ADC maps neither in pigs grafted with pMSCs or dogs following cMSC injections (Fig. 2C–F respectively). Quantitative assessment of signal intensity histograms of selected ROIs for both ipsilateral and contralateral hemispheres showed the accumulation of hypointense pixels on SWI in the ipsilateral hemisphere (Fig. 2C′,D′) compared to the contralateral hemisphere (Fig. 2C″,D″). Histogram did not show any changes in diffusion on ADC maps after cell transplantation (Fig. 2E′–F″). To examine whether the transplantation procedure led to chronic focal effects such as brain atrophy, measurements of hemispheric and ventricular symmetry were performed before surgery and a few months after cell transplantation. No statistically significant difference in symmetry has been detected before versus after therapy with GRPs (*P* = 0.86, *P* = 0.86 for left and right hemisphere respectively; *P* = 0.48 and *P* = 0.51 for left and right ventricle respectively; Fig. 2G) or MSCs (*P* = 0.76, *P* = 0.84 for left and right hemisphere respectively; *P* = 0.53, *P* = 0.55 for left and right ventricle respectively; Fig. 2H). Clinically, there was no evidence of focal damage, hemorrhage or stroke in T2w MRI neither in the dogs treated with GRPs (Fig. 2G) nor in those treated with MSCs (Fig. 2H).

### Histological detection of transplanted pMSCs in swine

**Figure 3:**
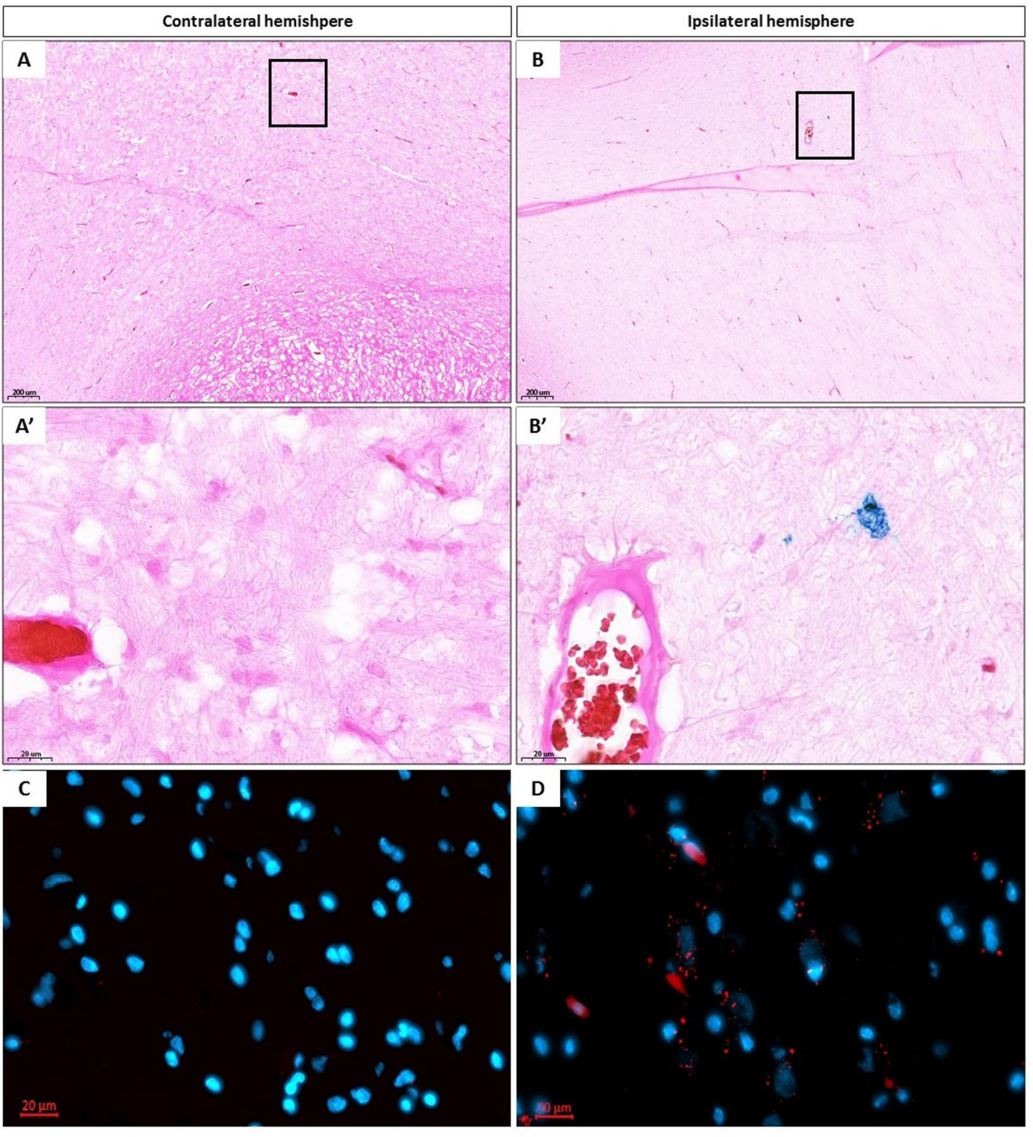
Histological evaluation. Prussian blue staining of brain of pigs after SPION pMSCs transplantation in contralateral [(**A**) ×5 magnification, (**A′**) ×40 magnification] and ipsilateral hemisphere [(**B**) ×5, (**B′**) ×40 magnification]. DAPI staining of brain of pigs after SPION pMSC transplantation in collateral [(**C**) ×40 magnification] and ipsilateral hemisphere [(**D**) ×40 magnification]. Red spots indicate iron oxide nanoparticles present in pMSCs.

Histopathological analysis confirmed the distribution of pMSCs in regions shown during interventional MRI—guided pMSC transplantation procedure. Prussian blue and immunofluorescence staining facilitated detection of iron oxide nanoparticles present in pMSCs in the ipsilateral hemisphere (Fig. 3B,B′ and D) and lack of positive cells in the contralateral hemisphere (Fig. 3A,A′ and C). This presence of cells only in the ipsilateral hemisphere further demonstrates that cells entering circulation do not re-enter the brain.

### Impact of cMSCs transplantation on gene expression in DM dogs

**Figure 4:**
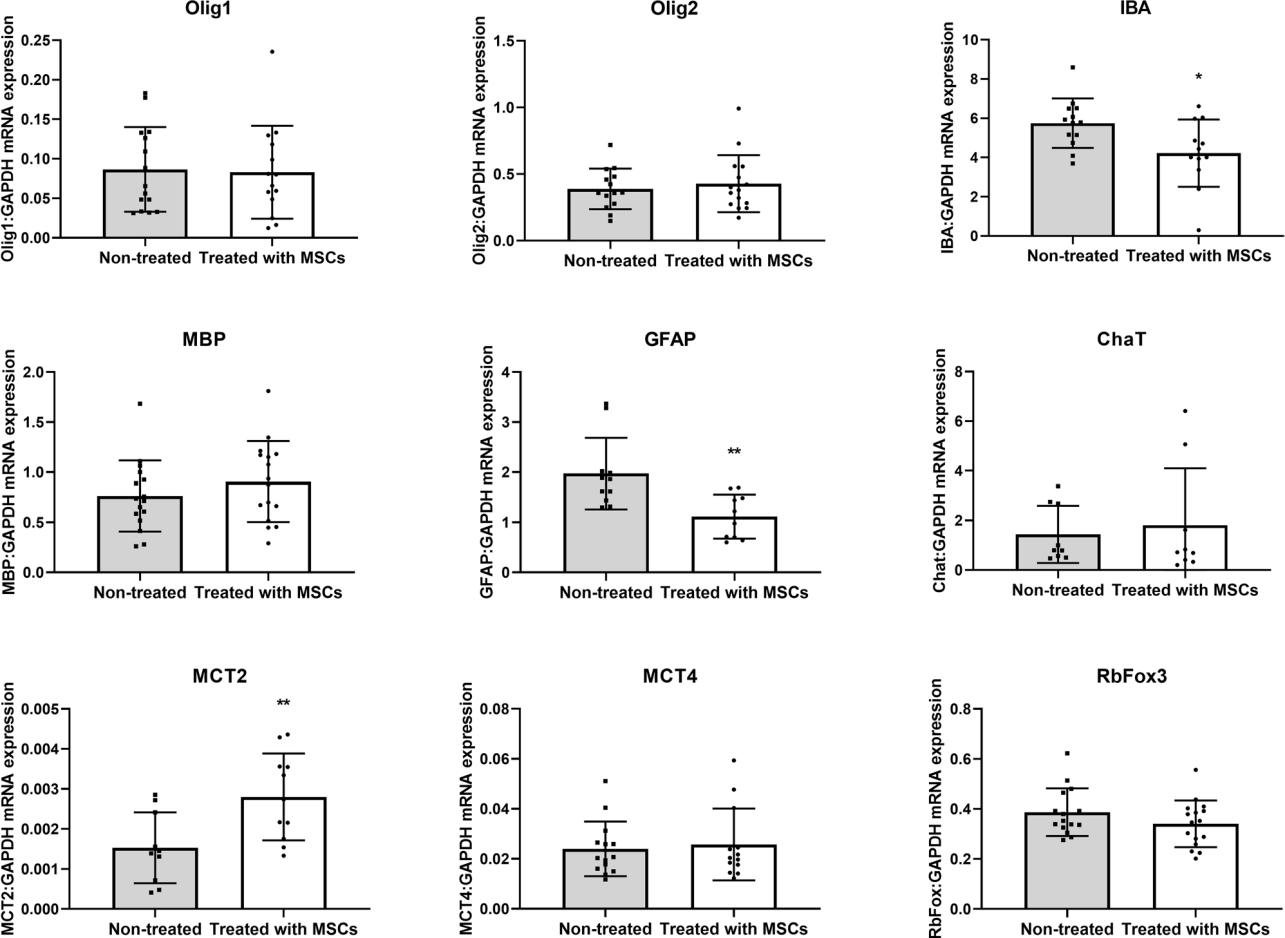
Real-time PCR analysis*.* Effect of canine MSCs transplantation on Olig1, Olig2, IBA, MBP, GFAP, Chat, MCT2, MCT4 and RBFox3 mRNA expression in the brains of DM dogs. Data are expressed as mean with standard deviation (SD) and differences were considered as statistically significant at the 95% confidence level (*P < 0.05, **P < 0.01).

With an effort to get an initial insight into potential therapeutic effects, we analyzed the expression of several transcripts in the brain that are of key importance in the context of neuronal and glial function and neurodegenerative processes. In dogs treated with MSCs, we observed decreased Iba-1 (*P* < 0.5) and GFAP (*P* < 0.01) mRNA expression in the ipsilateral compared to the contralateral (untreated) hemisphere. The MCT2 mRNA expression was increased in the hemisphere transplanted with cMSCs compared to the non-transplanted hemisphere (Fig. 4). We didn’t observe any change of Olig1 or Olig2, MBP, ChaT, MCT4, and RbFox3 mRNA expression after cMSC transplantation.

## Discussion

Over the past decade, there has been tremendous progress in the area of IA therapies, mainly fueled by advances in the treatment of ischemic stroke, but also in the delivery of therapeutics and stem cells to various organs^26,27^. The IA route was shown to be safe and cell targeting efficiency was superior compared to the IV route in animal models^8^. Cannulation of MCA via ICA in dogs was successful using 1.2 F microcatheter in contrast to Camstra et al.^28^ who reported frequent vasospasms while transitioning through tortuosities of ICA. We have demonstrated an excellent safety profile for the GRP delivery procedure in all transplanted animals. Thanks to dynamic, real-time MRI monitoring during cell infusion, it was possible to visualize the process of cell entrapment in the brain. This enables early intervention should any excessive accumulation and blockage of cerebral flow occur. The MRI scans did not show any abnormalities indicating microhemorrhages or stroke right after the transplantation or even after several months of follow up. However, the accumulation of GRP cells in the brain was very low. Most of the cells perfused away from the brain just after the infusion was completed. These results were in agreement with Lundberg et al.^29^, who showed poor human neural progenitors (hNPCs) and rat NPC recruitment in rat brains after IA transplantation in contrast to human MSCs. To address this problem, cells need to be either sorted for those with high expression of adhesion molecules^30^ or engineered to artificially induce expression of adhesion receptors^31,32^.

As the second class of widely used therapeutic cells, we used MSCs, which are larger in size and well known for their broad-based therapeutic mechanisms and high expression of adhesion molecules^33^. We previously utilized labeling and tracking of MSCs after transplantation in large animals^11^, and here we used this approach to examine the safety of the method and efficacy of pMSCs’ brain colonization in naive pigs.

To date, several studies of MSCs transplanted IA revealed adverse effects including reduced cerebral blood flow (CBF), increased mortality, and neurological impairment^34^. Given the fact that MSCs are significant, a key role in maintaining CBF and avoiding stroke is optimizing the velocity of administration and cell dose. Rat studies showed that the treatment of MSCs administered IA had a direct effect on reducing CBF^35^. Moreover, even low infusion velocity of MSCs was associated with many complications like necrotic cell-loss and blood brain barrier leakage^36^. Similarly, the dose of MSCs as well as pericyte-progenitor cells infused IA had a critical role in microembolic complications in dogs^37,38^. Considering the potential difficulties regarding CBF after the IA administration of cells demonstrated in small and large animal models, we decided to evaluate the safety of the method first on pigs.

We did not observe any adverse effects following transplantation, and brain accumulation of pMSCs was better. This outcome, based on dynamic MR imaging, was further confirmed by histology, where we found an abundance of pMSCs in the areas which correlated with MRI scans obtained on the day of administration. Subsequent experiments with transplantation of cMSCs in dogs with DM also showed excellent safety despite robust cell retention following transplantation. Much higher retention compared to GRPs is likely due to the larger size of MSCs or by the higher expression of adhesion molecules^32,39^. In comparison, the primary focus of this work demonstrating the feasibility and safety of the IA delivery focal localization of injected cells in the ipsilateral hemisphere offered a unique opportunity to gain insight into potential therapeutic effects. With cGRPs not accumulating in the brain in sufficient numbers, we focused this analysis on cMSCs. Based on tissues of dogs that succumb to DM, we observed a reduction in transcripts for IBA and GFAP, indicating the anti-inflammatory effect of cMSCs^40^. Because degenerative myelopathy, like ALS in humans, is correlated with inflammation, mainly present in the CNS^41^, MSC transplantation via the IA route can be an excellent anti-inflammatory therapy in such diseases, as confirmed in our current research.

To summarize, our study demonstrated that IA injection of stem cells is safe and, in the case of MSCs, results in excellent cell accumulation in the brain making it an attractive route for delivery in neurodegenerative diseases like ALS. It represents an essential next step towards clinical use in ALS patients. However, for cells not equipped with an appropriate set of adhesion molecules, taking full advantage of this route will require cell engineering.

## Material and methods

### Isolation of pMSCs

The pMSCs were isolated as described previously^4^. Bone marrow (BM) was aspirated from juvenile pig iliac crest using a syringe containing 10 ml PBS with 200 µl heparin (Polfa, Warsaw, Poland). The BM was diluted with PBS (Gibco, Gaithersburg, USA) in a ratio of 1:2. Next, the mixture was layered on a Ficoll-Paque Plus (Sigma Aldrich, LGC Standards, UK) and centrifuged at 310 g at room temperature (RT) for 25 min. A ring of mononuclear cells was collected into a new tube with 20 ml of PBS and centrifuged at 413 g for 10 min in RT. Next, the cell pellet was washed twice with PBS. Cells were suspended in the BMMSC medium, (Gibco) and plated on 25 ml flasks at 37 °C in a humified incubator with 5% CO_2_. Cultured cells were maintained for 15–20 days (2–3 passages), harvested with Accutase (Gibco), cryopreserved in a freezing medium (Sigma Aldrich), and stored in vapor-phase liquid nitrogen.

### Isolation of cGRPs and cMSCs

cGRPs were isolated from canine fetuses between E32-37, as described previously^4^. cMSCs were isolated from five, healthy, mixed breed gyps tested negative for SOD1 mutation and the cells were pooled. The lower abdomen adipose tissue was isolated during the sterilization of the bitch, with the agreement of the owner. The adipose tissue was washed with PBS (Gibco) 3 times and treated with Collagenase II (Gibco) for 3 h at 37 °C. Next, the collagenase was neutralized with serum from the donor and centrifuged at 426 g for 10 min in RT. The pellet was then passed through cell strainers, washed with PBS, and centrifuged at 334 g for 10 min twice. Then, the cells were counted and cryopreserved in a freezing medium (Sigma Aldrich), and stored in vapor-phase liquid nitrogen until used.

### Animals

All animal experiments were approved by the University of Warmia and Mazury’s ethics committee. The ARRIVE guidelines was followed for all animal studies. Experiments were performed according to the EU Directive 2010/63/EU as well as Poland Act 2015/01/15 “Act on the protection of animals used for scientific or educational purposes”. Additionally, for transplantation procedures on dogs, written permission of dog owners was obtained.

#### Experimental animals (pigs)

Three juvenile, Large White domestic pigs (40 kg, both sexes) were used. At least two weeks before transplantation, animals were acclimated to the new environment and human presence to minimize the stress associated with the experiment. Pigs had access to water and food ad libitum.

#### Experimental animals (dogs)

Eight dogs were recruited from veterinary clinics, for the study based on neurological exam identifying symptoms of DM and genetic test confirming mutations in the SOD-1 gene. For immunosuppression, dogs received daily Equoral (cyclosporin 8–10 mg/kg, Teva Pharmaceuticals, Poland). Cyclosporin concentration in blood was measured and maintained at 100–150 ng/ml.

### Cell preparation for the transplantation procedure

All types of cells (cGRP, cMSC, pMSC) were thawed three days before transplantation. The cGRPs were plated in cell culture flasks coated with PLL/Laminin in GRP medium (DMEM/F12 supplemented with N2 and B27, bovine serum albumin, heparin, and β-FGF; Gibco, Peprotech). MSCs were plated on uncoated, culture flasks within (DMEM supplemented with 10% of FBS; Gibco). One day before transplantation, the medium was supplemented with 25 µg/ml SPION particles (Molday, BioPal, Worcester, MA, USA) for labeling and detection in MRI. The next day cells were washed with phosphate buffered saline (PBS), harvested, and centrifuged at 222 g for 5 min. For transplantation, the cells pellet was suspended in PBS (4–5 × 10^6^/5 ml).

### MRI-guided intra-arterial transplantation of pMSCs

During the transplantation procedure, animals were anesthetized with a combination of sevoflurane (2%) and propofol (3 mg/kg/h). Anesthetized pigs were placed under C-arm, where 5F sheath (TerumoMedical) was introduced percutaneously to the femoral artery. Next, an angiographic catheter (5F, Balton) was navigated under X-ray guidance to the common carotid artery over hydrophilic 0.35 guidewire (Merit Laureate, Merit Medical). Microcatheter (UltraFlow HPC Flow Directed Micro Catheter, ev3) was introduced over a micro guidewire (Hybrid 0.008", Balt) to the ascending pharyngeal artery with the tip placed proximally to the rete mirabile. Continuous flow flushing of heparinized (5000 U/l) saline was maintained to avoid occlusion of the microcatheter. The rate of infusion adjusted during contrast agent pre-injection ranged 1.5–4 ml/min with 2.5 ml/min being defined as optimal. Cells were transplanted under real-time MRI with the acquisition of dynamic T2*-weighted images (GE-EPI TR/TE:1750/30 ms). The MRI protocol also included T2 (TR/TE = 5851/83 ms) and T1 (TR/TE = 1111.4/10 ms) scans prior and post cell delivery, SWI (TE/TR = 20/28) and diffusion-weighted with ADC mapping (TE/TR = 104/4800; b-value was 0, 500 and 1000) after transplantation to assess if the procedure and the cells caused brain damage.

### MRI-guided intra-arterial transplantation of cGRPs and cMSCs

Eight dogs (5–12 years) of various breeds (1 German Shepherd, 3 Hovawart, 3 Bernese mountain, one crossbred dog) were divided into two groups: (1) with GRP transplantation (three animals; one female and two males; age 5–11 years) and (2) with cMSC transplantation (five animals; one female and four males; age 8–9 years). During the procedure, animals were anesthetized as described above. The 5-French introducer (ProCardia TerumoMedical) was inserted into the femoral artery and navigated to the carotid artery using fluoroscopy. Next, a 1.2-French microcatheter was advanced into the middle cerebral artery under roadmap guidance as described previously^42^. Then, dogs were transferred to a 3 T MRI scanner and positioned supine. MRI protocol was the same as that for pig studies with T2*-weighted dynamic imaging of cells biodistribution in real-time and T2, T1 scans prior and post cell delivery. Cells were transplanted with the same range of infusion rate as in pig experiment. Just after the procedure during recovery from anesthesia, dogs were hydrated with Ringer's fluid and were returned to their owners for recovery. The follow up monthly visits included blood analysis and neurological examination. MRI was done six months post-transplantation. For calculations of a brain covered by cells, Horos software was used.

### Tissue samples

Brain tissues of pigs were collected immediately after slaughter, 24 h after pMSC transplantation, fixed in 4% paraformaldehyde, for 48 h at 4 °C, cryo-protected in 30% sucrose until sunk, frozen on dry ice for 5 min and kept at − 80 °C for histological analysis. Brains from dogs that succumbed to the disease were harvested immediately after euthanasia (2 and 7 months post-transplantation), and each hemisphere was divided into two parts from which one was protected in liquid nitrogen and stored at − 80 °C for gene expression analysis and the other was protected for histology as described above. The rest of the transplanted dogs are still alive.

### Histopathological analysis

For the detection of SPION labeled cells, Prussian Blue staining was performed. Porcine brain tissues were dried in RT for 10 min and washed in distilled water. The next sections were flooded in a mixture of equal parts of aqueous potassium ferrocyanide (5%) and aqueous hydrochloric acid (5%) for 30 min. Then sections were washed in distilled water three times for 5 min and counterstained with an aqueous solution of eosin for 1 min. After rinsing in distilled water, sections were dehydrated, cleared, and mounted in DPX (Sigma Aldrich). Sections were analyzed on a scanner (3D Histech).

### Total RNA extraction and reverse transcription

Canine brain tissues form dogs that succumbed to disease were used for total RNA extraction using a commercial kit (A&A Biotechnology, Poland). The quality of RNA and its concentration was measured using a NanoDrop 1000 spectrophotometer (Thermo Fisher Scientific Inc., DE, USA). Subsequently, reverse transcription reactions were done using the Reverse Transcription System Kit (Applied Biosystems, CA, USA). Two types of RT controls were used, one without RNA and another in the absence of the reverse transcriptase.

### Real-time polymerase chain reaction (RT-PCR)

The cDNA obtained was used for real-time quantitative PCR analysis using the Applied Biosystems 7900HT Fast Real-Time PCR System (Applied Biosystems, USA). Each sample contained 3 µL (36 ng) cDNA, 1.5 µL RNAse-free water (Promega, USA), 5 µL TaqMan Universal MasterMix II (Life Technologies, USA) and 0.5 µL TaqMan assays (*MBP* -Cf02641118_m1, *GFAP*-Cf02655693_g1, *IBA*-Cf02653363_m1, *Olig1*-Cf02685151_s1, *MCT4*-Cf02702665_g1, *ChaT*-Cf02724445_m1, *RbFox3*-Cf02658562_m1, *GAPDH*-Cf04419463_gH, *β-actin-Cf04931159_m1*, *cyclophilin* (Cf02665149_m1. *Olig2* and *MCT2* were made to order; Life Technologies, USA). All PCR runs were performed as described previously^4^. Data obtained from the RT-PCR were normalized using the ratio of mRNA examined to the *GAPDH* mRNA. Quantification of gene expression was performed using the comparative CT method.

### Statistical analysis

Statistical analyses were performed using GraphPad Prism 8.0 (GraphPad Software, Inc, San Diego, CA). The distribution of normality was evaluated with Kolmogorov–Smirnov test. The student's t-test was used to determine the difference in mRNA expression. The two-way ANOVA followed by Bonferroni's post hoc test was used to determine the size of hemispheres and ventricles and to assess differences between brain areas covered by MSC and GRP. All numerical data are presented as mean with standard deviation (SD) and differences were considered as statistically significant at the 95% confidence level (*P* < 0.05).

## Supplementary Information

Supplementary Information

## Data Availability

The datasets used and/or analyzed during the current study are available from the corresponding author on reasonable request.

## References

[1] Uccelli A, Laroni A, Freedman MS (2013). Mesenchymal stem cells as treatment for MS—progress to date. Multiple Scler..

[2] Silani V, Cova L, Corbo M, Ciammola A, Polli E (2004). Stem-cell therapy for amyotrophic lateral sclerosis. Lancet.

[3] Golubczyk D (2019). The role of glia in canine degenerative myelopathy: relevance to human amyotrophic lateral sclerosis. Mol. Neurobiol..

[4] Malysz-Cymborska I (2018). MRI-guided intrathecal transplantation of hydrogel-embedded glial progenitors in large animals. Sci. Rep..

[5] Kurtz A (2008). Mesenchymal stem cell delivery routes and fate. Int. J. Stem Cells.

[6] Labusca L, Herea DD, Mashayekhi K (2018). Stem cells as delivery vehicles for regenerative medicine-challenges and perspectives. World J. Stem Cells.

[7] Fischer UM (2009). Pulmonary passage is a major obstacle for intravenous stem cell delivery: the pulmonary first-pass effect. Stem Cells Dev..

[8] Walczak P (2008). Dual-modality monitoring of targeted intraarterial delivery of mesenchymal stem cells after transient ischemia. Stroke.

[9] Qin H (2017). rabbit model of human gliomas: implications for intra-arterial drug delivery. PLoS ONE.

[10] Janowski M, Walczak P, Pearl MS (2016). Predicting and optimizing the territory of blood–brain barrier opening by superselective intra-arterial cerebral infusion under dynamic susceptibility contrast MRI guidance. J. Cereb. Blood Flow Metab. Off. J. Int. Soc. Cerebr. Blood Flow Metab..

[11] Walczak P (2017). Real-time MRI for precise and predictable intra-arterial stem cell delivery to the central nervous system. J. Cereb. Blood Flow Metab. Off. J. Int. Soc. Cerebr. Blood Flow Metab..

[12] McGoldrick P, Joyce PI, Fisher EM, Greensmith L (2013). Rodent models of amyotrophic lateral sclerosis. Biochim. Biophys. Acta.

[13] Lyczek A (2017). Transplanted human glial-restricted progenitors can rescue the survival of dysmyelinated mice independent of the production of mature, compact myelin. Exp. Neurol..

[14] Herrmann AM (2019). Large animals in neurointerventional research: a systematic review on models, techniques and their application in endovascular procedures for stroke, aneurysms and vascular malformations. J. Cereb. Blood Flow Metab. Off. J. Int. Soc. Cerebr. Blood Flow Metab..

[15] Savitz SI, Baron JC, Fisher M, Consortium SX (2019). Stroke treatment academic industry roundtable X: brain cytoprotection therapies in the reperfusion era. Stroke.

[16] Boltze J (2019). Stem cells as an emerging paradigm in stroke 4: advancing and accelerating preclinical research. Stroke.

[17] Rao MS, Mayer-Proschel M (1997). Glial-restricted precursors are derived from multipotent neuroepithelial stem cells. Dev. Biol..

[18] Lepore AC, Fischer I (2005). Lineage-restricted neural precursors survive, migrate, and differentiate following transplantation into the injured adult spinal cord. Exp. Neurol..

[19] Lepore AC (2008). Focal transplantation-based astrocyte replacement is neuroprotective in a model of motor neuron disease. Nat. Neurosci..

[20] Walczak P (2011). Human glial-restricted progenitors survive, proliferate, and preserve electrophysiological function in rats with focal inflammatory spinal cord demyelination. Glia.

[21] Lepore AC (2011). Human glial-restricted progenitor transplantation into cervical spinal cord of the SOD1 mouse model of ALS. PLoS ONE.

[22] Karussis D (2010). Safety and immunological effects of mesenchymal stem cell transplantation in patients with multiple sclerosis and amyotrophic lateral sclerosis. Arch. Neurol..

[23] Zhou YY, Yamamoto Y, Xiao ZD, Ochiya T (2019). The immunomodulatory functions of mesenchymal stromal/stem cells mediated via paracrine activity. J. Clin. Med..

[24] Srivastava AK, Bulte CA, Shats I, Walczak P, Bulte JW (2016). Co-transplantation of syngeneic mesenchymal stem cells improves survival of allogeneic glial-restricted precursors in mouse brain. Exp. Neurol..

[25] Boltze J (2015). The dark side of the force—constraints and complications of cell therapies for stroke. Front. Neurol..

[26] Misra V, Ritchie MM, Stone LL, Low WC, Janardhan V (2012). Stem cell therapy in ischemic stroke: role of IV and intra-arterial therapy. Neurology.

[27] Saraf J (2019). Intra-arterial stem cell therapy modulates neuronal calcineurin and confers neuroprotection after ischemic stroke. Int. J. Neurosci..

[28] Camstra KM (2020). Canine model for selective and superselective cerebral intra-arterial therapy testing. Neurointervention.

[29] Lundberg J (2012). Targeted intra-arterial transplantation of stem cells to the injured CNS is more effective than intravenous administration: engraftment is dependent on cell type and adhesion molecule expression. Cell Transpl..

[30] Chua JY (2011). Intra-arterial injection of neural stem cells using a microneedle technique does not cause microembolic strokes. J. Cereb. Blood Flow Metab..

[31] Gorelik M (2012). Use of MR cell tracking to evaluate targeting of glial precursor cells to inflammatory tissue by exploiting the very late antigen-4 docking receptor. Radiology.

[32] Jablonska A (2018). Overexpression of VLA-4 in glial-restricted precursors enhances their endothelial docking and induces diapedesis in a mouse stroke model. J. Cereb. Blood Flow Metab..

[33] Nitzsche F (2017). Concise review: MSC adhesion cascade-insights into homing and transendothelial migration. Stem Cells.

[34] Guzman R, Janowski M, Walczak P (2018). Intra-arterial delivery of cell therapies for stroke. Stroke.

[35] Janowski M (2013). Cell size and velocity of injection are major determinants of the safety of intracarotid stem cell transplantation. J. Cerebr. Blood Flow Metab..

[36] Cui LL (2015). The cerebral embolism evoked by intra-arterial delivery of allogeneic bone marrow mesenchymal stem cells in rats is related to cell dose and infusion velocity. Stem Cell Res. Ther..

[37] Lu SS (2013). In vivo MR imaging of intraarterially delivered magnetically labeled mesenchymal stem cells in a canine stroke model. PLoS ONE.

[38] Youn SW (2015). Feasibility and safety of intra-arterial pericyte progenitor cell delivery following mannitol-induced transient blood brain barrier opening in a canine model. Cell Transpl..

[39] Karp JM, Leng Teo GS (2009). Mesenchymal stem cell homing: the devil is in the details. Cell Stem Cell.

[40] Lo Sicco C (2017). Mesenchymal stem cell-derived extracellular vesicles as mediators of anti-inflammatory effects: endorsement of macrophage polarization. Stem Cells Transl. Med..

[41] Lovett MC (2014). Quantitative assessment of hsp70, IL-1beta and TNF-alpha in the spinal cord of dogs with E40K SOD1-associated degenerative myelopathy. Vet. J..

[42] Golubczyk D (2020). Endovascular model of ischemic stroke in swine guided by real-time MRI. Sci. Rep..

