# Supplementary material for: Intra-arterial transplantation of stem cells in large animals as a minimally-invasive strategy for the treatment of disseminated neurodegeneration
Source: Sci Rep. 2021 Mar 22;11:6581. doi: 10.1038/s41598-021-85820-3 (PMC7985204; doi:10.1038/s41598-021-85820-3)
Supplement: Supplementary file 1 — Supplementary Information [file 41598_2021_85820_MOESM1_ESM.docx]

**Intra-arterial transplantation of stem cells in large animals as a minimally-invasive strategy for the treatment of disseminated neurodegeneration**

Izabela Malysz-Cymborska^1#^, Dominika Golubczyk^1#^, Lukasz Kalkowski^1^, Joanna Kwiatkowska^1^, Michal Zawadzki^2^, Joanna Głodek^3^, Piotr Holak^3^, Joanna Sanford^4^, Kamila Milewska^1^, Zbigniew Adamiak^3^, Piotr Walczak^5,^ and Miroslaw Janowski^5^

^1^ Dept of Neurosurgery, School of Medicine, Collegium Medicum, University of Warmia and Mazury, Olsztyn, Poland

^2^ Central Clinical Hospital of Ministry of the Interior and Administration in Warsaw, Poland

^3^ Dept of Surgery and Radiology, Faculty of Veterinary Medicine, University of Warmia and Mazury, Olsztyn, Poland

^4^ Sanford Biotech, Warsaw, Poland

^5^ Center for Advanced Imaging Research and Department of Diagnostic Radiology and Nuclear Medicine, University of Maryland School of Medicine, Baltimore, MD, USA

#These authors contributed equally to this work

***To whom correspondence should be addressed:**

**Izabela Malysz-Cymborska**

Department of Neurosurgery

School of Medicine

University of Warmia and Mazury, Olsztyn, Poland

ORCID iD 0000-0003-4192-6726

[i.m.cymborska@gmail.com](mailto:i.m.cymborska@gmail.com)

**
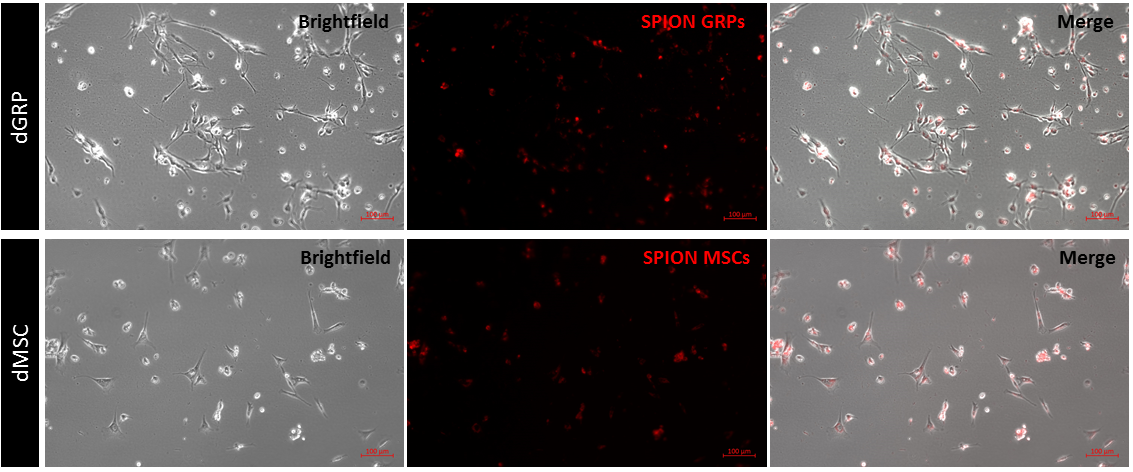
Supplementary Figure 1.**  ***Light microscopy of cells before transplantation.*** Images of cGRPs (upper panel) and cMSCs (bottom panel) labeled with SPION for transplantation (10x magnification).
